# Transcriptome-Based Identification of Genes Responding to the Organophosphate Pesticide Phosmet in *Danio rerio*

**DOI:** 10.3390/genes12111738

**Published:** 2021-10-29

**Authors:** Bala Murali Krishna Vasamsetti, Kyongmi Chon, Juyeong Kim, Jin-A Oh, Chang-Young Yoon, Hong-Hyun Park

**Affiliations:** Toxicity and Risk Assessment Division, Department of Agro-Food Safety and Crop Protection, National Institute of Agricultural Sciences, Rural Development Administration, Wanju-gun 55365, Korea; vbmk84@gmail.com (B.M.K.V.); kjy.sara@gmail.com (J.K.); oja5074@korea.kr (J.-A.O.); evermoo2600@korea.kr (C.-Y.Y.); honghyunpark@korea.kr (H.-H.P.)

**Keywords:** pesticide toxicity, phosmet, transcriptome analysis, zebrafish

## Abstract

Organophosphate pesticides (OPPs) are one of the most widely used insecticides. OPPs exert their neurotoxic effects by inhibiting acetylcholine esterase (AChE). Most of the gross developmental abnormalities observed in OPP-treated fish, on the other hand, may not be explained solely by AChE inhibition. To understand the overall molecular mechanisms involved in OPP toxicity, we used the zebrafish (ZF) model. We exposed ZF embryos to an OPP, phosmet, for 96 h, and then analyzed developmental abnormalities and performed whole transcriptome analysis. Phenotypic abnormalities, such as bradycardia, spine curvature, and growth retardation, were observed in phosmet-treated ZF (PTZF). Whole transcriptome analysis revealed 2190 differentially expressed genes (DEGs), with 822 and 1368 significantly up-and downregulated genes, respectively. System process and sensory and visual perception were among the top biological pathways affected by phosmet toxicity. Kyoto Encyclopedia of Genes and Genomes (KEGG) pathway analysis revealed significant enrichment of metabolic pathways, calcium signaling pathway, regulation of actin cytoskeleton, cardiac muscle contraction, drug metabolism–other enzymes, and phototransduction. Quantitative real-time PCR results of six DEGs agreed with the sequencing data expression profile trend. Our findings provide insights into the consequences of phosmet exposure in ZF, as well as an estimate of the potential risk of OPPs to off-target species.

## 1. Introduction

Pesticides improve crop productivity and quality by killing insects, fungi, and weeds; however, their off-target effects on aquatic flora and fauna have raised concerns about pesticide-related human health and environmental risks [1]. 

Organophosphates are one of the most widely used insecticides. In addition to their use in agriculture, organophosphates are used as chemical warfare agents, and flame retardants [2,3]. In fact, organophosphate pesticides (OPPs) are extremely toxic to humans and account for most self-poisoning deaths in developing countries [4,5]. The consequences of OPPs on humans are primarily linked to neurological disorders [6]. Children were reported to exhibit neurological and developmental abnormalities after being exposed to indoor OPPs [7]. OPPs were reported to induce developmental and behavioral abnormalities in animals, birds, reptiles, amphibians, and fish [8]. Because OPPs are increasingly being used in developing countries [9], it is essential to better understand their off-target effects.

Phosmet is a non-systemic, phthalimide-derived OPP used to control moths, aphids, mites, suckers, and fruit flies in plants and animals [10]. It is widely used on fruit crops, ornamental, cattle, sheep, goats, and pigs [10]. According to the California Department of pesticide regulation, an average of 0.4 million pounds of phosmet was applied in California based on 2000–2018 statistics [11]. Oral ingestion or inhalation of phosmet has been shown to induce severe toxicity in rat acute toxicity studies [10] while adversely impacting normal development and ossification in murine models [12]. Surface water runoff and spray drift are expected to be the major routes of exposure for phosmet. According to the surface water database from the California Department of pesticide regulation, the maximum concentration of phosmet was 0.63 μg/L in 2010 [13]. Acute phosmet exposure is extremely toxic to freshwater fish and invertebrates, and chronic exposure has been shown to have negative impacts on freshwater fish growth and survival [13]. Phosmet is toxic to *Daphnia magna* (48 h EC_50_: 0.0056 mg/L) and other fish such as bluegill sunfish (96 h LC_50_: 0.07 mg/L), channel catfish (96 h LC_50_: 11.0 mg/L), fathead minnow (96 h LC_50_: 7.3 mg/L), and rainbow trout (96 h LC_50_: 0.241 mg/L) [13]. 

Zebrafish (ZF) is an established alternative to other animal models owing to its higher fertility rate, large brood size, short life cycle, and transparent embryos [14]. ZF has been widely used in the toxicological assessment of pesticides and other chemicals and is considered a suitable model for acute toxicity studies [14,15]. Notably, ZF shares 87% gene homology with humans, which indicates high conservation of signaling cascade components between humans and ZF [16]. Fish exposed to OPPs had lower levels of acetylcholine esterase (AChE) and showed behavioral impairments, such as abnormal swimming and reduced predatory escape response [17,18]. In addition to behavioral issues, OPPs have been linked to various other malformations in fish, including growth retardation, body curvature, vision impairment, and cardiac abnormalities [19,20,21]. Recently, we evaluated phosmet toxicity in the early stages of ZF development and found various developmental defects, including body curvature, deformed heart, and growth retardation, in addition to behavioral abnormalities [19]. Despite extensive research into the developmental toxicity of OPPs in ZF, the mechanism of how these defects occur is unclear.

The inhibition of AChE, which results in the accumulation of acetylcholine (ACh) in central and peripheral synapses, altering neurotransmission, has been identified as one of the mechanisms involved in OPP toxicity [22,23]. Deformities, such as growth inhibition, edema formation, and craniofacial and spinal deformities observed in OPP-treated fish [19,20,21], may not be a consequence of AChE inhibition alone. A comparison of molecular, neurobehavioral, and biochemical responses to OPPs suggests that AChE may be an insignificant biomarker for OPP toxicity in ZF [24]. On the other hand, a wide range of OPP-induced toxic mechanisms, including disruption of neurotransmitter metabolism, induction of oxidative stress, muscle exhaustion, calcium dysregulation, and induction of immunological and inflammatory responses, have been proposed in ZF [25,26]. Therefore, it is necessary to understand OPP-induced toxicity at the molecular level.

Understanding the molecular mechanism involved in pesticide-induced toxicity is challenging. Next-generation sequencing (NGS) has gained popularity due to its ability to detect unique genes with high throughput. Accordingly, ZF transcriptome studies have successfully been used to understand the molecular insights of antibiotic-induced [27], pesticide-induced [28], and nanoparticle-induced [29] toxicity. Our study aims to document the molecular responses of OPP-induced toxicity at the transcriptome level, as well as to identify critical genes and signaling pathways altered in response to toxicity. Pesticide-treated ZF samples were used as a model; briefly, 2 hpf ZF embryos were treated with phosmet until 96 hpf, and high-throughput RNA sequencing was performed. We provide information on the important genes and biological pathways altered in parallel with the developmental observations in phosmet-treated ZF (PTZF).

## 2. Results

### 2.1. Effects of Phosmet on ZF Development

**Figure 1:**
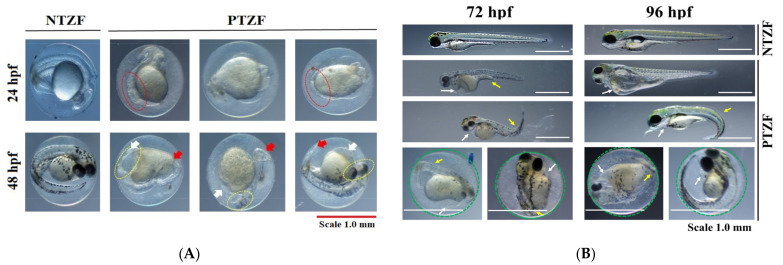
Representative images showing phosmet-induced deformities: (**A**) 24 hpf (upper panel) and 48 hpf (lower panel); (**B**) 72 hpf (left side panel) and 96 hpf (right side panel). Red dotted circles, abnormal somites; yellow dotted circles, abnormal eye formation or reduced retinal pigmentation; red arrow, malformed tails; white arrow, pericardial edema; yellow arrow, spine curvature; green dotted circle, unhatched embryos. NTZF, non-treated zebrafish; PTZF, phosmet-treated zebrafish. Scale = 1.0 mm.

Phosmet has been reported to cause various developmental abnormalities in ZF embryos and larvae [19]. Figure 1 shows the developmental deformities of PTZF at 24, 48, 72, and 96 hpf. A wide range of developmental defects, including abnormal somites, reduced retinal pigmentation, edema, body-axis curvature, and sensory abnormalities, was observed in PTZF at a dose of 8.0 mg/L (Table 1).

**Figure 2:**
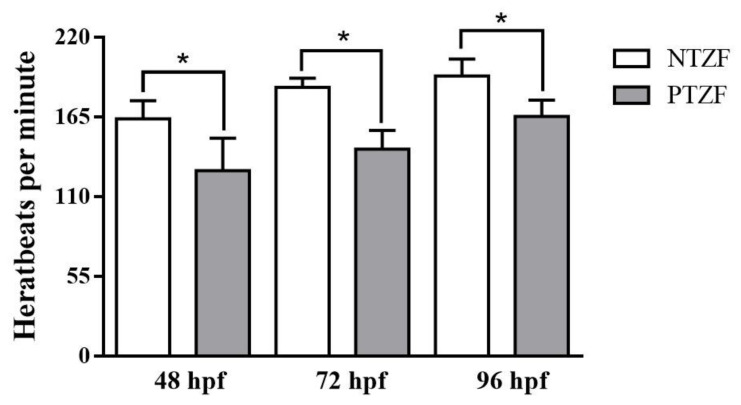
Average heartbeats per minute at 48, 72, and 96 hpf. NTZF, non-treated zebrafish; PTZF, phosmet-treated zebrafish. The results are mean ± SD (*n* = 3). Statistical significance was evaluated using an unpaired *t*-test. * (*p* < 0.0001).

The cardiac defects in PTZF ranged from an extremely slow heart rate to subtle changes in blood flow (Figure 2 and Table 1). The average heart rates of the controls were 163.6 ± 12.2, 185.2 ± 6.3, and 193.2 ± 11.5 beats per minute (bpm) at 48, 72, and 96 hpf, respectively (Figure 2). PTZF, with 127.8 ± 22.0, 142.8 ± 12.6, and 165.2 ± 11.1 bpm at 48, 72, and 96 hpf, respectively, had a lower heart rate than non-treated ZF (NTZF). Pericardial edema (PE) was also observed in PTZF (Table 1). Approximately 20% of PTZF showed symptoms of PE at 24 hpf, and by 72 hpf, the incidence of PE increased to 62%. In ZF with PEs, reduced blood flow and hyperemia were also frequently observed (Table 1). These findings suggest that phosmet affects cardiac development and function in ZF.

NTZF quickly escaped upon touch, whereas PTZF displayed an abnormal touch-evoked escape response (TEER) (Table 1). When PTZF were gently touched on the head or tail, they reacted in a mild to non-reactive manner. Most NTZF quickly identified the object placed in their field of binocular vision and escaped quickly to the opposite corners of the Petri dish. However, PTZF failed to identify the object and the subsequent vision-mediated escape response (VMER) (Table 1), suggesting abnormal sensory responses in PTZF.

**Figure 3:**
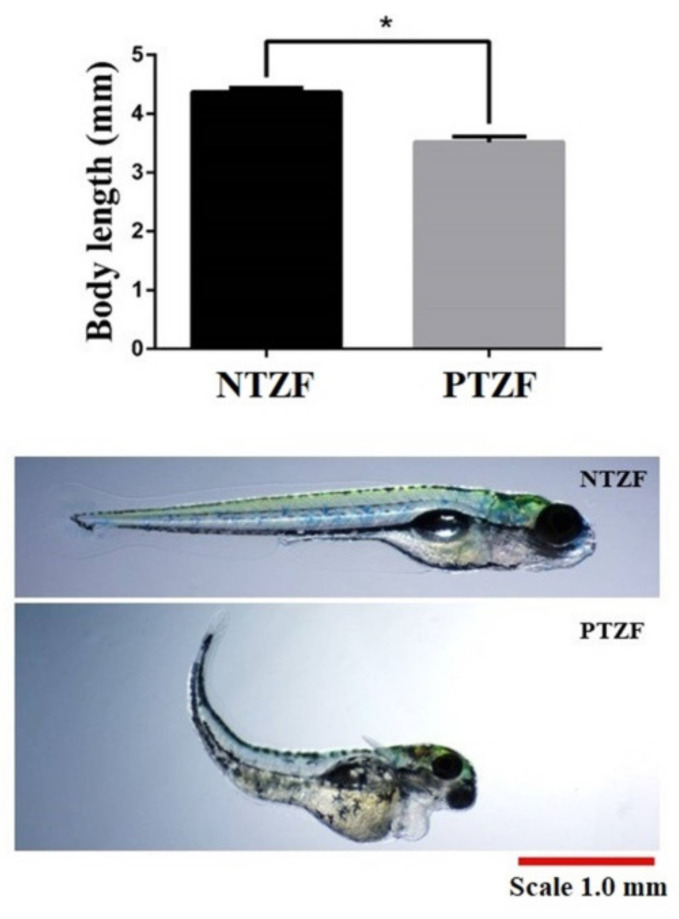
The graph represents the average body length at 144 hpf. Representative images show the body length at 144 hpf. NTZF, non-treated zebrafish; PTZF, phosmet-treated zebrafish. Scale = 1.0 mm. The results are mean ± SD (*n* = 4). Statistical significance was evaluated using an unpaired *t*-test. * (*p* < 0.001).

Phosmet decreased the overall growth of ZF (Figure 3). At 144 hpf, PTZF displayed a 20% shorter body length than NTZF. NTZF and PTZF had an average body length of 4.4 ± 0.1 mm and 3.5 ± 0.1 mm, respectively.

### 2.2. Transcriptome Analysis

**Figure 4:**
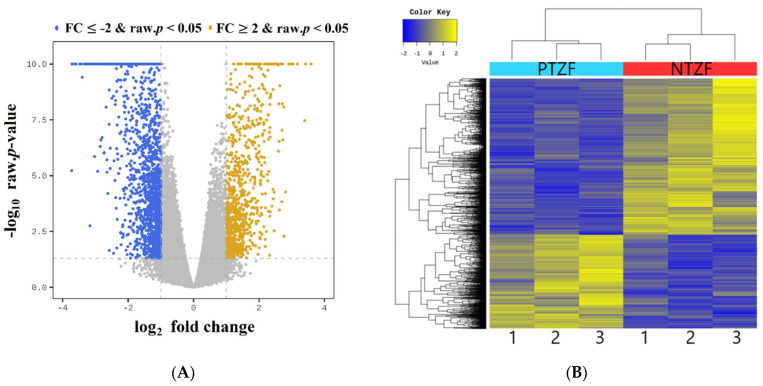
(**A**) The volcano plot for differentially expressed genes (DEGs) of phosmet-treated compared and non-treated zebrafish (NTZF). Individual colored dots indicate upregulated (yellow, FDR < 0.05 and FC ≥ 2) and downregulated (blue, FDR < 0.05 and FC ≤ −2) genes in the phosmet-treated group; (**B**) heat map of differential expression in NTZF vs. PTZF. Heat map of one-way hierarchical clustering using the *Z*-score for normalized values (log2 based).

To determine the effect of phosmet on global transcription in ZF, high-throughput whole-transcriptome analysis was performed, with three replicates for each treatment (control (*n* = 3) and 8.0 mg/L of phosmet (*n* = 3), 96 hpf). The details of Illumina sequencing data are shown in Table 2. For all biological replicates, Illumina-based sequencing data produced 399,175, and 608 clean reads. More than 95% of clean reads had quality scores above the Q 30 threshold, and a mean of 81.92% of clean reads was uniquely mapped to the ZF reference genome (GRCz11). The GC content of the clean reads ranged from 43.60% to 53.27%. Volcano plots were constructed by integrating both *p*-value and fold change of each transcript to illustrate the general scattering of the transcripts and filter differentially expressed genes (DEGs) between PTZF and NTZF (Figure 4A). DEGs were selected with cutoff values of fold change ≥ 2 and adjusted false discovery rate (FDR) correction *p*-value < 0.05. A total of 2190 DEGs were identified in PTZF, of which 62.46% (1368) were downregulated and 37.53% (822) were upregulated and were annotated, as shown in the volcano plot (Figure 4A). Figure 4B shows the heat map of one-way hierarchical clustering analysis using the *Z*-score for normalized value (log2-based). On the basis of transcriptome analysis, the top downregulated (FC < −5) genes in response to phosmet treatment include microRNA 1-1 (*mir-1-1*), transglutaminase 1 like 3 (*tgm1l3*), potassium voltage-gated channel, shaker-related, subfamily, member 6 a (*kcna6a*), crystallin, alpha A (*cryaa*), crystallin beta-gamma X (*crybgx)*, glutaminase 2a (liver, mitochondrial)- transcript variant X2 (*gls2a)*, and solute carrier family 10 (sodium/bile acid cotransporter), member 1, transcript variant X2 (*slc10a1*). The top upregulated (FC > 5) genes include glutathione S-transferase pi 2 (*gstp2*), cytochrome P450, family 1, subfamily A (*cyp1a*), heat shock cognate 70-kd protein, like (*hsp70l*), heat shock cognate 70-kd protein, tandem duplicate 3 (*hsp70.3*), heat shock cognate 70-kd protein, tandem duplicate 1, transcript variant X2 (*hsp70.1*), ictacalcin 2 (*icn2*), cofilin 1 (non-muscle)-like (*cfl1l*), glial cells missing homolog 2 (Drosophila) (*gcm2*), guanine nucleotide-binding protein (G protein), alpha 14 (*gna14*), and caspase b (*caspb*) (Table S1).

### 2.3. Gene Ontology Term Enrichment

**Figure 5:**
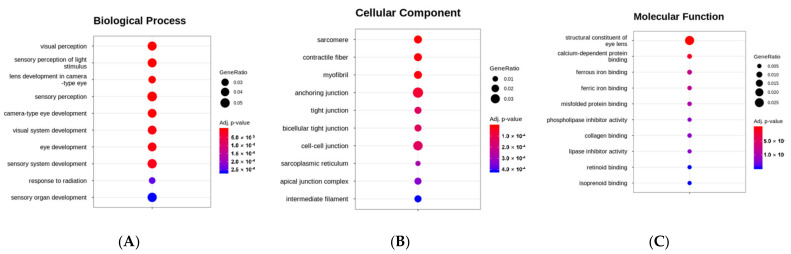
Top 10 enriched functional gene ontology terms in biological process (BP) (**A**), cellular component (CC) (**B**), and molecular function (MF) (**C**) of differentially expressed genes.

Next, we performed gene ontology (GO) enrichment analysis of significant DEGs to functionally classify the genes in three subcategories: cellular component (CC), biological process (BP), and molecular function (MF). Figure 5 shows the top 10 GO terms in each category after phosmet treatment. Significantly enriched terms for “BP” were associated with system process, visual perception, nervous system processes, and sensory perception (Figure 5A); for “CC,” with membrane and extracellular region (Figure 5B); for “MF,” with structural molecule activity, transporter activity, and ion binding (Figure 5C).

### 2.4. Functional Annotation of DEGs

**Figure 6:**
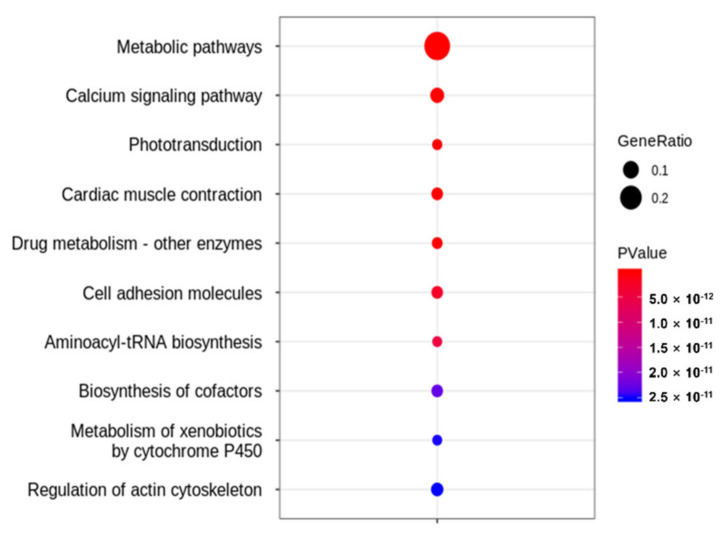
Top 10 KEGG pathways in phosmet-treated zebrafish compared with non-treated zebrafish.

To identify enriched pathways upon phosmet treatment, DEG data were evaluated using the Kyoto Encyclopedia of Genes and Genomes (KEGG) pathway enrichment analysis. The results revealed an enrichment of 71 KEGG pathways (*p* ≤ 0.05) clustered into six groups—namely, metabolism (34 pathways), genetic information processing (3 pathways), environmental processing (10 pathways), cellular processes (12 pathways), organismal systems (11 pathways), and human diseases (1 pathway) (Figure S1). The top 10 highly enriched KEGG pathways (*p* ≤ 0.001) included metabolic pathways (dre01100), calcium signaling pathways (dre04020), phototransduction (dre04744), cardiac muscle contraction (dre04260), drug metabolism—other enzymes (dre00983), cell adhesion molecules (dre04514), aminoacyl-tRNA biosynthesis (dre00970), biosynthesis of cofactors (dre01240), metabolism of xenobiotics by cytochrome P450 (dre00980), and regulation of actin cytoskeleton (dre04810) (Figure 6). DEGs up- or downregulated in the top 10 KEGG pathways are shown in Table 3.

### 2.5. qPCR Validation of Randomly Selected DEGs 

Using qPCR, we examined the transcript levels of six randomly selected DEGs that showed upregulation or downregulation trends. All genes had the same expression pattern as seen in RNA sequencing (Table 4).

## 3. Discussion

Given the high toxicity of OPPs, understanding the molecular mechanisms affecting their non-specific targets is critical. Pesticide-induced toxicity is undoubtedly complicated and can disrupt cellular homeostasis [30]. In this study, we used next-generation RNA sequencing, which is one of the promising techniques for comprehensive transcriptome analysis [31], to translate and understand phosmet-induced toxicity at the molecular level in the ZF model. When comparing the transcriptome of PTZF to that of NTZF, 2,190 DEGs were obtained, with 822 and 1368 significantly up- and downregulated DEGs, respectively (File S1). The identified DEGs were enriched in pathways related to metabolism, muscle development and contraction, ion transport and signaling, and neuron development and signaling (Figure 5 and Figure 6). 

AChE suppression is a major toxic mechanism of OPPs, and AChE measurement is the most frequently used biomarker for environmental pollution with OPPs [30]. AChE inhibition leads to ACh buildup at the synapse, which induces a wide range of behavioral defects in fish, including abnormal swimming and a lack of responsiveness to touch and visual cues [22]. Consistently, decreased AChE expression was observed in PTZF (File S1) and in treatments with other OPPs, including envoy 50 SC [32], chlorpyrifos [33], and diazinon [24], all of which have been reported to induce behavioral and developmental abnormalities in ZF. By contrast, some OPPs, such as dichlorvos, malathion, and methyl-parathion, did not suppress AChE, but induced deformities in ZF [21]. Richendrfer and Créton revealed that relatively low quantities of OPPs can influence ZF behavior without altering AChE activity [34]. These findings imply that in addition to, or instead of, suppressing AChE, OPPs affect other molecular mechanisms that regulate development and behavior. Based on the obtained transcriptome data (Figure 5 and Figure 6), it is reasonable to speculate that the phosmet can affect many pathways in addition to AChE inhibition. Further research is required to distinguish AChE inhibition from the pathways described.

Gross developmental abnormalities such as curved spines, edema formation, and growth retardation were the most significant in PTZF (Figure 1, Figure 3, and Table 1). The OPP, dichlorvos, which induces developmental abnormalities in ZF [20], altered several genes involved in energy metabolism and oxidative stress responses [35]. In ZF, metabolism is primarily responsible for development into adult fish [36]; therefore, maintaining metabolic homeostasis is critical. With 151 DEGs, “metabolic pathways” (dre01100) was the top enriched term in KEGG pathway analysis (Figure 6), suggesting that phosmet exposure can affect multiple metabolic pathways in early developmental stages.

Among metabolic pathways, oxidative phosphorylation (OxPhos) (dre00190) was significantly enriched in PTZF (Figure S1). OPPs were reported to adversely affect mitochondrial complexes I, II, III, IV, and V, mitochondrial ATP production, and mitochondrial membrane potential [37]. Several OxPhos-related genes, such as the subunits of NADH dehydrogenase (*ND1, ND2, ND3, ND4, ND4L, ND5*), NADH dehydrogenase (ubiquinone) 1 alpha subcomplexes, (*ndufa1, ndufa2,* and *ndufa4*), succinate dehydrogenase (ubiquinone) membrane anchor subunit *(SDHD)*, ubiquinol–cytochrome c reductase cytochrome b subunit *(Cytb)*, and type H^+^-transporting ATPase subunit a *(ATP6)* were downregulated in PTZF (File S1). These findings suggest that mitochondrial toxicity is one of the potential mechanisms of phosmet toxicity; thus, a deeper understanding of how OPPs induce mitochondrial toxicity might lead to potential treatment strategies.

Oxidative stress is involved in OPP-mediated toxicity [38]. Reduced expression of cytochrome c oxidase (CcO) was linked to increased production of reactive oxygen species [39]; CcO inhibition in ZF induced developmental abnormalities and increased apoptosis in the hindbrain and neural tube [40]. The downregulation of several (CcO)-related genes (SCO2 cytochrome c oxidase assembly protein (*sco2*), cytochrome c oxidase subunits (*cox1, cox2, cox3, cox20,* and *cox16*)*,* and cytochrome c, somatic b (*cycsb*)) (File S1) and developmental deformities were observed in PTZF (Table 1). Glutathione metabolism (dre00480) was one of the enriched KEGG terms in PTZF (Figure S1). Notably, glutathione S-transferase (GST)-related genes (glutathione S-transferase pi 1 (*gstp1*)*,* glutathione S-transferase pi 2 (*gstp2*)*,* glutathione S-transferase mu tandem duplicate 3 (*gstm.3*)*,* and microsomal glutathione S-transferase 1.2 (*mgst1.2*) were upregulated (File S1). GSTs are one of the most important enzymes in phase II cellular detoxification, which detoxifies xenobiotics and products of oxidative stress [41]. Such alterations imply that phosmet exposure leads to oxidative stress.

The calcium signaling pathway (dre04020) was found to be significantly enriched in PTZF (Figure 6), implying a disruption of Ca^2+^ homeostasis. Being a secondary messenger, Ca^2+^ plays vital roles in somite formation, eye development, brain partitioning, and heart formation, thus the establishment of the basic vertebrate body plan of ZF [42]. Calcium signaling has also been implicated in early muscle development, neuronal induction, regulation of neural tube closure, cardiogenesis, and phagocytosis at wound sites [43]. Inhibiting calcium signaling during embryonic development induced several developmental abnormalities in ZF, including cyclopia and tail deformities [44]. Consistently, in this study, PTZF exhibited gross developmental and behavioral abnormalities, such as increased incidence of edema and eye defects and decreased response to touch (Table 1).

PTZF failed to establish cardiac function (Table 1 and Figure 2), which is consistent with our previous results [19], where phosmet induced a dose-dependent increase in PE, bradycardia, and abnormalities in blood flow. OPPs, such as diazinon [45], dichlorvos [21], and sumithion [20], were also reported as cardiac toxicants. Cardiac problems could arise from defects in heart development and abnormalities in the conduction system or the contractile apparatus [46]. PE is a sign of abnormal cardiac development, whereas heart rate variations are indicators of abnormal cardiac function [47]. Alterations in the rate or pattern of blood flow are an additional sign of abnormal heart function. Our functional analysis revealed enrichment of numerous cardiac-related pathways (Figure 5 and Figure 6) that are crucial for maintaining the structural and functional integrity of the heart, indicating that phosmet exposure affected both heart development and function. Cardiac muscle contraction (dre04260), ECM-receptor interaction (dre04512), adrenergic signaling in cardiomyocytes (dre04261), calcium signaling (dre04020), regulation of actin cytoskeleton (dre04810), and cytokine–cytokine receptor interactions (dre04060) are a few indicators of such alterations.

The top downregulated gene in PTZF, *mir1-1*, was implicated in the regulation of cardiac contraction and embryonic angiogenesis in ZF [48]. In ZF, the downregulation of *mir-1* is associated with the dysregulation of muscle gene expression, thereby affecting sarcomeric actin assembly. Knocking out *mir-1* led to the failure of embryonic blood vessel formation. In fact, when observed under the microscope, PTZF showed malformed blood vessels, resulting in abnormal blood flow (Table 1). RNA sequencing analysis also showed that the expression of *tnnt2, ttn, heg1, mybpc2, trdn*, and *itga10* were downregulated in PTZF compared with NTZF (Table S2). Silent heart mutants lacking the cardiac troponin T gene (*tnnt2*) failed to form myofibrils [49], and in the absence of titin, nascent myofibrils developed normally, but higher-order sarcomeric structures were absent [50]. Moreover, heart development protein with EGF-like domains 1 (*heg1*) mutants exhibited atrial and ventricular enlargement, reduced heart rate and blood flow, and PE [51]. Myosin binding protein C (encoded by *mybpc*) is critical for the maintenance of sarcomere integrity; the knockdown of *mybpc* caused cardiac hypertrophy, diastolic heart failure, and PE [52]. In mice, *Trdn* knockout led to impaired heart muscle excitation–contraction coupling [53]. Integrins (*itga10*) are cell surface receptors that play a significant role in mechanotransduction, and therefore the heart rate [54]. In comparison to NTZF, PTZF exhibited a 10-fold overexpression of *cyp1a* (Table S2). In the ZF heart failure model, Cyp1 suppression reduced doxorubicin-induced cardiomyopathy [55]. These findings, consistent with deformity data such as pericardial edema symptoms and a decreased heart rate (Table 1 and Figure 2), suggest that phosmet can interfere with cardiogenesis and the cardiac conduction system.

The cytoplasmic Ca^2+^ in the heart area increases several folds during cardiogenesis [56], and therefore, perturbation of calcium signaling interferes with normal cardiogenesis and subsequently, cardiac function. For instance, the downregulation of *pkd2*, as observed in PTZF (Table S2), encodes polycystin-2, a non-selective calcium-regulated cation channel expressed on the endoplasmic/sarcoplasmic membrane, resulting in reduced cardiac contractile function [57]. There is evidence that mitochondrial dysfunction is linked to cardiovascular manifestations [58] and indeed, mitochondrial inhibitors induced PE, bradycardia, and arrhythmia in the ZF model [59]. Thus, the cardiovascular abnormalities of PTZF can be attributed in part to mitochondrial dysfunction and calcium signaling perturbations during development.

PTZF had smaller eyes (Figure 1), reduced retinal pigmentation (by 48 hpf), and no VEER, compared with NTZF (Table 1). These findings are consistent with those of our previous study, where phosmet-induced ocular toxicities were found to be dose-dependent [19]. In other studies, OPPs have been shown to have deleterious effects on the visual system [60]. PTZF exhibited a pattern of downregulation in fatty acid-binding protein 11b (*fabp11b*), a critical component in fatty acid metabolism in developing eyes [61]. Another study found that *fabp11b* was downregulated in response to cypermethrin [28]. A recent transcriptome-based analysis of ZF found that OPP poisoning changed the expression of numerous genes involved in ocular development [28]. One of the most enriched pathways in PTZF was phototransduction (dre04744) (Figure 6). In PTZF, the cone opsin genes, including long (*opn1lw1* (opsin 1 (cone pigments), long-wave-sensitive, 1)*, opn1lw2* (opsin 1 (cone pigments), long-wave-sensitive, 2)), medium (*opn1mw2* (opsin 1 (cone pigments), medium-wave-sensitive, 2), *opn1mw2* (opsin 1 (cone pigments), medium-wave-sensitive, 2)), and short (*opn1sw1* (opsin 1 (cone pigments), short-wave-sensitive 1)*, opn1sw2* (opsin 1 (cone pigments), short-wave-sensitive 1)) wavelength-sensitive opsins, and rhodopsin (*rho*) were downregulated (File S1), suggesting vision impairment. The downregulated pattern of certain recoverins (*rcvrnb, rcvrn2, rcvrn3*) and cone-specific kinase (*grk7a*) in PTZF (File S1) suggests altered photoresponse recovery [62]. Crystallins, which constitute a substantial part of the refractive structure of the vertebrate eye lens and play a critical role in embryonic lens development, were downregulated in PTZF [63]. A single-cell transcriptome analysis study of ZF showed that crystallins (*cryaa* (crystallin, alpha A) and *crybgx* (crystallin beta-gamma X)) are exclusively expressed in lens fiber cells starting at 2 dpf [64]. In our study, the biological process linked with visual system development and perception was among the top GO terms (Figure 5), indicating that phosmet triggers ocular toxicity.

According to our KEGG analysis, the aminoacyl-tRNA biosynthesis pathway was highly enriched in PTZF (Figure 6), indicating that protein biosynthesis was disrupted. Most genes encoding aminoacyl tRNA synthetase (ARS), which ligate tRNAs to their corresponding amino acids, were downregulated (Table 2). Thus, the growth retardation observed in PTZF (Figure 3) is attributable to abnormal protein synthesis. In addition to their important involvement in protein biosynthesis, ARSs also required in other biological processes. Recently, Inoue et al. reported that leucyl-tRNA synthetase deficiency induces autophagy in ZF [65]. Loss of leucyl-tRNA synthetases leads to liver failure in ZF [66]. Waldron et al. reported that histidyl-tRNA synthetases are essential for the proliferation and survival of neuronal progenitors during development [67].

Genes related to the biosynthesis of co-factors, such as *nqo1*(NAD(P)H dehydrogenase, quinone 1), *pts* (6-pyruvoyltetrahydropterin synthase), *gch2* (GTP cyclohydrolase 2), and *nme2a* (NME/NM23 nucleoside diphosphate kinase 2a), were downregulated upon pesticide treatment (Table 2). *NQO1* is involved in cardiovascular disease in humans [68]. Reduced expression of pyruvoyltetrahydropterin synthase (encoded by *pts*) was associated with hyperphenylalaninemia and dopamine and serotonin depletion in the central nervous system [69]. Abnormal expression of *gch2* (GTP cyclohydrolase 2) in the ZF *camembert* (*cmm*) recessive mutant resulted in pigmentation defects during embryonic and larval stages [70]. Desvignes et al. reported that the *nme* (NME/NM23 nucleoside diphosphate kinase 2a) gene family is required for ZF oogenesis and early embryonic development [71]. UDP glucuronosyltransferases (UGT) (*ugt5a1, ugt5a4, ugt1a5, ugt1a7, ugt1a1, ugt2a4, ugt1a2,* and *ugt1a6*) were upregulated in PTZF (File S1). UGTs convert hydrophobic toxicants to hydrophilic toxicants through glucuronidation and are therefore involved in xenobiotic detoxification, drug clearance, and endobiotic metabolism [72]. 

Considering that ZF shares a high degree of gene homology with humans [15], understanding the mechanism of pesticide toxicity in ZF can provide insights into its consequences in humans. The ZF model can produce in vivo toxicity assessments within weeks, which is much faster than that for mammalian testing [73]. Animal toxicology studies frequently uncover effects that demand further investigation to elucidate the causes, which is both expensive and time consuming. The existing screening methods in ZF enable understanding very early details of off-target effects, and when combined with transcriptome analysis, understanding the potential mechanism and crosstalk between mechanisms. In addition, the impact of pesticides on the cardiovascular system, central nervous system, digestive tract, and visual functions and their proconvulsant potential can be assessed at the molecular level. Therefore, ZF-based technology should be recognized as an important prefilter to aid in the identification of the safest candidates for pesticide development and the determination of pesticide poisoning treatments. This study uncovered various molecular mechanisms affected by pesticide toxicity, thereby demonstrating the feasibility of using novel techniques for cumulative risk assessment.

## 4. Materials and Methods

### 4.1. Ethics Statement

All animal experiments were performed in accordance with the guidelines for the care and use of laboratory animals, approved by the Animal Ethics Committee of the National Institute of Agricultural Sciences, Rural Development Administration, Republic of Korea (NAS-202102). 

### 4.2. ZF Maintenance and Embryo Collection

ZF (AB strain) were maintained in a glass aquarium filled with dechlorinated tap water and equipped with a recirculating filtration system. The photoperiod was 14 h light/10 h dark and the temperature was 25 ± 1 °C. ZF were fed 2–3 times a day with live brine shrimp (INVE aquaculture, Dendermonde, Belgium), blood worms (Hikari Bio-pure, Himeji, Japan), and dry flake food (Top meal, Tabia, Korea). The protocols used for ZF embryo collection and cleaning were as described [70,74]. Briefly, five females and five males were transferred to a nylon mesh, which was installed in a plastic aquarium filled with water, and covered with a transparent lid. On the next day, the fertilized eggs were collected 30 min after the light was turned on and washed three times in E3 medium (290 mg of sodium chloride, 8.3 mg of potassium chloride, 48 mg of calcium chloride, 81.5 mg of magnesium chloride, and 100 μL of 1% methylene blue per 1 L of distilled water).

### 4.3. Pesticide Treatment and Deformities Scoring

The final test concentration of phosmet [2-dimethoxyphosphinothioylthiomethyl) isoindoline 1,3-dione] (#36195, purity 98%, Sigma Aldrich, St. Louis, MO, USA) was prepared in E3 buffer. The test dose of 8.0 mg/L was chosen based on our prior work [19] as having twice the EC_50_ value (4.38 ± 0.18 mg/L) of phosmet to ZF, ensuring that most scored abnormalities are present in all collected embryos (RNA sequencing samples). Phosmet and E3 medium exposures were started at approximately 2.0 hpf in an incubator with a constant temperature of 26 ± 1 °C and under dark conditions. The test solutions were replaced every 24 h. The experiments were conducted in 24-well plates and repeated twice with two 24-well plates (each plate containing 20 embryos) per test condition. Deformities were scored under a stereoscopic microscope (Stemi 508, Zeiss, Germany). The methods of deformities scoring and percentage calculation were as described [18,74]. Abnormalities in the somites and tail detachment, as well as symptoms of edema, were scored at 24 hpf. Accumulation of retina pigmentation, abnormal blood flow (caudal region), and hyperemia were scored at 48 hpf. At 72 hpf, hatching, pericardial, and yolk sac edema were scored. Body curvature and hatching were scored at 96 hpf. The deformity percentage was calculated as the ratio of embryos that showed deformity overall live embryos at the time point. Statistical significance was evaluated using an unpaired *t*-test. 

### 4.4. Heartbeat Survey

A heartbeat survey was conducted at 48, 72, and 96 hpf. Embryos or larvae were anesthetized with a final concentration of 10 mg/L tricaine (ethyl 3-aminobenzoate methanesulfonate) (Sigma-Aldrich, St. Louis, MO, USA) just before being observed. The heartbeats of embryos or larvae were counted for 20 s under a stereoscopic microscope at room temperature of 26 ± 1 °C. The counts were adjusted to bpm and presented. A total of 10 embryos from each test condition at each time point were analyzed, and the experiment was performed 3 times. Statistical significance was evaluated using an unpaired *t*-test.

### 4.5. TEER and VMER

TEER was monitored at 96 hpf by gently touching the head and tail of ZF with a flexible plastic wire (1 mm). VMER was monitored at 96 hpf by gently moving a 1 mm diameter plastic tip in the field of binocular vision. The experiments were repeated three times, each time with ten embryos per test condition. Statistical significance was evaluated using an unpaired *t*-test.

### 4.6. Body Length Survey 

A body length survey was conducted at 144 hpf. Pictures were captured with a stereomicroscope. Body length (from the mouth tip to the end of the tail fin) was obtained using OptiView 3.7 software (Korealabtech, Seongnam, Korea). The experiments were repeated twice with 12 embryos per test condition. Statistical significance was evaluated using an unpaired *t*-test. 

### 4.7. RNA Sequencing Preparations

Three phosmet-treated and three non-treated ZF samples were collected by snap freezing in liquid nitrogen and were stored at −80 °C until use. Each sample contained approximately 60 embryos or larvae. Total RNA was extracted using the QIAzol® Lysis Reagent (QIAGEN, Cat. No. 79306, Hilden, Germany) and RNeasy® Mini Kit (QIAGEN, Cat. No. 74106, Hilden, Germany). Total RNA concentration was quantified using Quant-IT RiboGreen (Invitrogen, #R11490, Waltham, MA, USA), and RNA integrity was assessed using the TapeStation RNA screen tape (Agilent, Santa Clara, CA, USA). RNA preparations with a RIN greater than 7.0 were used to construct the RNA libraries. Illumina TruSeq Stranded Total RNA Library Prep Gold Kit (Illumina, Inc., San Diego, CA, USA, #20020599) was used to prepare the independent libraries using 0.5 μg of total RNA from each sample. The Ribo-Zero rRNA Removal Kit (Human/Mouse/Rat Gold) (Illumina, Inc., San Diego, CA, USA) was used to remove rRNA from total RNA. Then, the remaining mRNA was fragmented into small pieces using divalent cations under high temperatures. Using SuperScript II reverse transcriptase (Invitrogen, #18064014, Waltham, MA, USA) and random primers, the cleaved RNA fragments were transcribed into the first strand of cDNA. Second-strand cDNA synthesis was then performed using DNA polymerase I, RNase H, and dUTP. These cDNA fragments were then subjected to end repair, followed by the addition of a single A base and adapter ligation. After purifying the products, the final cDNA libraries were prepared by PCR. The KAPA Library Quantification Kits for Illumina Sequencing Platforms (KAPA BIOSYSTEMS, #KK4854, Bath, UK) were used to quantify the libraries, and TapeStation D1000 ScreenTape (Agilent Technologies, # 5067-5582, Santa Clara, CA, United States) was used to qualify libraries. The indexed libraries were subjected to paired-end (2 × 100 bp) sequencing by Macrogen Incorporated on an Illumina NovaSeq platform (Illumina, Inc., San Diego, CA, USA).

### 4.8. Transcriptome Alignment and Analysis of DEGs

DEGs were selected (nbinomWaldTest using DESeq2) with a cut-off of absolute log2 fold change ≥ 2 and a *p*-value of 0.05 by comparing PTZF and NTZF. Further analyses were performed by using the obtained DEGs.

### 4.9. Gene Ontology and Pathway Enrichment Analysis

Gene Ontology Enrichment analysis was performed using the g:Profiler tool (https://biit.cs.ut.ee/gprofiler/, accessed on 13 January 2021) for DEGs. Pathway enrichment analysis based on KEGG Pathways (http://www.kegg.jp/kegg/pathway.html, accessed on 14 January 2021)) was performed for DEGs.

### 4.10. Quantitative Real-Time PCR

Total RNA was extracted from three phosmet-treated and three non-treated ZF samples using the RNeasy Mini Kit (Bioneer, Daejon, South Korea), and analyzed with the Nanodrop 2000 (Thermo Fisher Scientific, MA, USA). RNA was reverse transcribed into cDNA using the ReverTra Ace™ qPCR RT master mix with gDNA remover (Toyobo, Osaka, Japan). Quantitative real-time PCR was performed on the CFX96 Dx real-time PCR detection system (Biorad, CA, USA) using the TOPreal™ qPCR 2X premix (Enzynomics, Daejon, Korea). Each sample was analyzed in triplicate and the average Ct values were used for fold change calculations. β-actin was used as a housekeeping gene. The 2^−ΔΔCT^ method was applied for the expression analysis. The list of primers used for qPCR is presented in Supplementary Table S1.

## 5. Conclusions

In conclusion, these results demonstrate that phosmet alters the expression of a plethora of genes, impacting fish overall growth and behavior. These findings primarily provided detailed information on the altered molecular pathways to phosmet that causes developmental toxicity in ZF. 

## Figures and Tables

**Table 1:** Percentage of deformities observed at 24, 48, 72, and 96 h after phosmet treatment.

Time	Deformity (%)	NTZF	PTZF
24 hpf	Mortality	0	17.50 ± 2.89 *
	Edema symptoms	2.50 ± 1.44	19.54 ± 7.11 *
	Abnormal somites	2.50 ± 1.25	69.76 ± 8.02 *
48 hpf	Mortality	0	32.50 ± 2.89 *
	Low retina pigment	0	63.07 ± 8.99 *
	Abnormal tail blood flow	1.25 ± 1.25	53.85 ± 7.97 *
	Hyperemia	0	44.22 ± 10.84 *
72 hpf	Mortality	2.50 ± 1.44	43.75 ± 6.29 *
	Unhatched embryos	1.25 ± 1.25	78.22 ± 6.05 *
	Pericardial edema	3.88 ± 1.30	61.63 ± 11.05 *
	Yolk sac edema	2.57 ± 1.48	48.11 ± 9.31 *
96 hpf	Mortality	2.50 ± 1.44	52.50 ± 3.23 *
	Unhatched embryos	0	82.29 ± 8.01 *
	Body curvature	0	80.21 ± 5.15 *
	VMER	25.00 ± 5.00	100.00 *
	TEER	10.00 ± 5.77	90.00 ± 5.77 *

Data are presented as the mean ± SD of four 24-well plates (20 embryos/plate) collected from two different experiments. Statistical significance was evaluated using an unpaired Student’s *t*-test. * Statistically significant (*p* < 0.005). NTZF, non-treated zebrafish; PTZF, phosmet-treated zebrafish; VMER, vision-mediated escape response; TEER, touch-evoked escape response. VEER and TEER data are presented as the mean ± SD of three experiments (10 embryos/experiment).

**Table 2:** Summary of sequence data generated for the transcriptome and quality filtering.

Samples	Total Reads	Clean Reads	Mapped Reads	Mapped Rate (%)	Q20 (%)	Q30 (%)	GC (%)
NTZF-1	72,783,012	71,783,716	60,458,452	84.22	98.91	96.05	43.62
NTZF-2	61,925,392	60,857,682	51,896,692	85.28	98.91	96.06	44.95
NTZF-3	60,878,020	59,726,314	51,603,485	86.4	98.85	95.87	46.00
PTZF-1	69,410,088	68,273,094	45,753,478	67.02	98.89	96.11	53.24
PTZF-2	66,557,046	65,493,004	55,524,220	84.78	98.92	96.13	49.44
PTZF-3	74,253,266	73,041,798	61,232,206	83.83	98.97	96.27	47.69

NTZF, non-treated zebrafish; PTZF, phosmet-treated zebrafish; Q20, Phred quality score 20; Q30, Phred quality score 30; GC, guanine–cytosine.

**Table 3:** A summary of KEGG pathway analysis for differentially expressed genes.

Term	Genes	*p*-Value	FDR
Metabolic pathways	gls2a, gls2b, gpx9, gda, hacd1, chia.2, gyg1b, tecrl2b, zgc:153896, elovl4b, zgc:153704, cyp7a1, zgc:103586, pgam2, pde6g, lpin1, ATP8, aoc1, LOC559107, ndufa4l2a, zgc:109982, pts, tyr, si:ch211-214p16.3, gch2, ampd1, mdh1ab, ND4L, tecrl2a, dhrs3a, atpv0e2, agxta, chs1, cyp21a2, aldoab, gal3st1a, ckmt2b, th2, si:dkey-78a14.5, dpm3, spam1, csgalnact1b, nat8l, ptges, atp5ia, nme2a, ND1, ND3, ND5, gpx8, ND2, si:dkey-78a14.4, aanat2, ndufa1, adssl1, si:ch211-217a12.1, ATP6, entpd8, alpi.2, tymp, ndufa4, elovl8a, entpd5a, zgc:112320, pigw, haao, CYTB, ckma, cel.1, gstk4, hao2, nme2b.2, pigh, pfkma, hpda, pnp4b, ND4, aldh3b2, smyd1a, b3gnt5a, mocs1, uox, st3gal7, si:ch211-106j24.1, pde6c, ndufa2, ugt5e1, fhit, sdhdb, acot19, agxtb, cyp2r1, COX3, COX1, COX2, tyrp1b, pde6d, acp5a *, sgpp2 *, galnt6 *, pik3c2g *, gal3st1b *, ugt5a1 *, atic *, glulc *, sqlea *, alp3 *, aldh3b1 *, p4ha1b *, paics *, elovl6l *, ethe1 *, plcd1a *, zgc:100864 *, zgc:154054 *, ugt1a5 *, cmasb *, aldob *, gstm.3 *, cyp24a1 *, ugt1a7 *, cbr1l *, zgc:123275 *, fut9d *, zgc:112332 *, b4galnt3b *, hao1 *, ugt1a1 *, mthfd1l *, si:ch73-337l15.2 *, ugt5a4 *, ptgs2b *, cmbl *, mgst1.2 *, rdh12l *, dao.1 *, zgc:163121 *, nansb *, si:ch211-276a23.5 *, cox4i1l *, pla2g4f.1 *, ugt1a2 *, ugt1a6 *, cyp3a65 *, cyp17a1 *, alas2 *, ptgs2a *, bhmt *, sqrdl *, nqo1 *, LOC565422 *, ptgis *, gstp1 *, ugt2a4 *, gstp2 *	1.14402 × 10^−53^	1.61306 × 10^−51^
Calcium signaling pathway	slc25a4, adrb2b, atp2a1l, mylk4b, stim2a, mylk4a, camk1gb, fgf4, ednraa, casq1a, trdn, tnnc2, p2rx5, avpr1aa, p2rx8, si:rp71-17i16.4, cacna1sb, casq2, calm1b, egf, ednrba, zgc:56235, casq1b, orai1a, si:dkey-247m21.3, fgf1a, p2rx3a, ryr3, atp2a1, phkg1b, tacr3a *, mcoln3a *, plcd1a *, p2rx1 *, si:dkey-251i10.1 *, cxcr4a *, ltb4r2a *, gna15.1 *, gna14 *	2.39485 × 10^−15^	1.68837 × 10^−13^
Phototransduction	opn1mw2, gngt1, opn1mw1, guca1c, gnat1, guca1d, pde6g, rhol, gnat2, rho, guca1e, rcvrnb, rcvrn2, grk7a, rcvrn3, calm1b, zgc:112320, cnga1	6.05641 × 10^−15^	2.84651 × 10^−13^
Cardiac muscle contraction	atp2a1l, si:ch211-139a5.9, cacng6b, zgc:163073, zgc:86725, cacng1a, trdn, tnnt2d, atp1b1a, tpm4b, tnnt2e, cacna1sb, casq2, atp1b2b, CYTB, atp1a3b, atp1b4, smyhc1, atp2a1, atp1b2a, COX3, COX1, COX2, cox4i1l *, atp1a1a.2 *	2.86244 × 10^−14^	1.00901 × 10^−12^
Drug metabolism—other enzymes	zgc:103586, si:dkey-78a14.5, nme2a, si:dkey-78a14.4, gstk4, nme2b.2, aldh3b2, ugt5e1, ugt5a1 *, tpmt.1 *, tpmt.2 *, aldh3b1 *, ugt1a5 *, gstm.3 *, ugt1a7 *, ugt1a1 *, ugt5a4 *, mgst1.2 *, ugt1a2 *, ugt1a6 *, gstp1 *, ugt2a4 *, gstp2 *	4.12652 × 10^−13^	1.16368 × 10^−11^
Cell adhesion molecules	cldn7a, cldn19, mpz, cldn5b, mag, si:ch211-286o17.1, cldnj, cdh15, itgb1a, cd99l2, cldnf, cdh1, cldnc, cldn8, itgb1b.1, oclnb, zgc:110333, cldn7b, cldnb, oclna, cldn1, cldne, si:ch211-95j8.5, cldni, zgc:136892	2.61473 × 10^−12^	6.14462 × 10^−11^
Aminoacyl-tRNA biosynthesis	trnS2, trnA, trnH, trnD, trnE, trnG, trnK, trnN, trnM, trnY, trnP, trnQ, trnC, trnR, trnI, trnL1, trnS1	4.82031 × 10^−12^	9.70947 × 10^−11^
Biosynthesis of cofactors	pts, gch2, dhrs3a, nme2a, adssl1, alpi.2, haao, nme2b.2, hpda, mocs1, ugt5e1, ugt5a1, alp3, ugt1a5, ugt1a7, zgc:112332, ugt1a1, mthfd1l, ugt5a4, rdh12l, ugt1a2, ugt1a6, alas2, nqo1, ugt2a4	2.2858 × 10^−11^	3.55548 × 10^−10^
Metabolism of xenobiotics by cytochrome P450	gstk4, aldh3b2, ugt5e1, ugt5a1,aldh3b1, ugt1a5, gstm.3, ugt1a7, cbr1l, ugt1a1, ugt5a4, mgst1.2, ugt1a2, ugt1a6, gstp1, ugt2a4, gstp2	2.50462 × 10^−11^	3.55548 × 10^−10^
Regulation of actin cytoskeleton	cfl2, cxcl12b, mylk4b, mylk4a, fgf4, mylpfb, cxcl12a, LOC101885790, si:dkey-44g17.6, itga10, egf, tmsb, scinla, pfn2l, itgb1a, fgf1a, si:ch73-116o1.2, brk1, itga2.2, itgb1b.1, cxcr4a, rac2, zgc:86896, myh9a, fn1b, itgb3a, gsnb, pfn1, cfl1l, zgc:101810	2.52162 ×10^−11^	3.55548 ×10^−10^

*: upregulated, normal text: downregulated, FDR: false discovery rate.

**Table 4:** qPCR validation of randomly selected up- and downregulated genes detected in transcriptome data.

Gene Symbol	Fold Change (RNA-Seq)	Fold Change (qPCR) (Mean ± SD)
*gna14*	6.13	4.17 ± 0.14
*fn1b*	4.14	2.13 ± 0.21
*gstp2*	12.09	5.15 ± 1.28
*gls2b*	−7.25	−4.40 ± 0.56
*cnga1*	−2.07	−2.84 ± 0.55
*adrb2b*	−4.27	−3.91 ± 1.02

## Data Availability

Data are contained within the article or Supplementary Materials.

## Supplementary Materials

The following are available online at https://www.mdpi.com/article/10.3390/genes12111738/s1, Figure S1: List of enriched pathways after phosmet treatment, Table S1: List of primers used for, Table S2: qPCRList of top upregulated and downregulated genes after phosmet treatment. File S1: List of DEGs after phosmet treatment.
