# Supplementary material for: Transcriptome-Based Identification of Genes Responding to the Organophosphate Pesticide Phosmet in Danio rerio
Source: Genes (Basel). 2021 Oct 29;12(11):1738. doi: 10.3390/genes12111738 (PMC8624534; doi:10.3390/genes12111738)
Supplement: Supplementary file 1 [file genes-12-01738-s001.zip › genes-1403670-supplementary/Supplementary material/Supplementary Table S1.pdf]

Table S1. List of oligo primers used in qPCR validation

Gene (Sequence ID)	Orientation	Oligo sequence
gna14 (NM_001003753.1)	Forward	ACAGGCGGAGTTCCAACAAA
	Reverse	CTCTGTGTAGCCCTTGCCAT
fn1b (NM_001013261.1)	Forward	CCTGGCGATGGCCATACTTT
	Reverse	CTGACCTGATGCTGGGTAGG
gstp2 (NM_001020513.1)	Forward	TCTCTTTGGACAGCTGCCTA
	Reverse	CGTTCTTTCCATACGCACCA
gls2b (NM_001083825.1)	Forward	ACAGAAACAAGTCGGTGGTG
	Reverse	GTCATAGTCCCTCAACTCCATATTC
cnga1 (XM_695944.9)	Forward	TTATCGGATGCGGTGTACCT
	Reverse	CGCGAAGCTTTTTCTGATCT
adrb2b (NM_001089471.2)	Forward	GAGTTTTGGACGGCTACGGA
	Reverse	CTGATAACGCAGAGGCCACA
β-actin	Forward	CGAGCTGTCTTCCCATCCA
	Reverse	TCACCAACGTAGCTGTCTTTCTG
