# Supplementary material for: Transcriptome-Based Identification of Genes Responding to the Organophosphate Pesticide Phosmet in Danio rerio
Source: Genes (Basel). 2021 Oct 29;12(11):1738. doi: 10.3390/genes12111738 (PMC8624534; doi:10.3390/genes12111738)
Supplement: Supplementary file 1 [file genes-12-01738-s001.zip › genes-1403670-supplementary/Supplementary material/Supplementary Table S2.pdf]

**Table S2.** List of top upregulated and top downregulated gens after phosmet treatment

| Transcript_ID | Gene_Symbol | Description | PTZF/NTZF.fc |
|---|---|---|---|
| NM_001020513 | gstp2 | glutathione S-transferase pi 2 | **12.09** |
| NM_131879 | cyp1a | cytochrome P450, family 1, subfamily A | **10.69** |
| NM_001017803 | zgc:111983 | zgc:111983 | **10.55** |
| NM_001113589 | hsp70l | heat shock cognate 70-kd protein, like | **9.03** |
| NM_131397 | hsp70.3 | heat shock cognate 70-kd protein, tandem duplicate 3 | **8.20** |
| XM_002667249 | si:cabz01007807.1 | si:cabz01007807.1 | **7.94** |
| NM_001252649 | si:ch211-117m20.5 | si:ch211-117m20.5 | **7.63** |
| XM_003198110,XM_005163922 | hsp70.1 | heat shock cognate 70-kd protein, tandem duplicate 1, transcript variant X2 | **7.62** |
| NM_001082957 | icn2 | ictacalcin 2 | **7.55** |
| NM_001291900 | si:ch211-195b11.3 | si:ch211-195b11.3 | **7.53** |
| NM_001007772 | zgc:101810 | zgc:101810 | **7.51** |
| NM_001020586 | mpeg1.2 | macrophage expressed 1, tandem duplicate 2 | **7.01** |
| XM_001334593 | LOC100002043 | uncharacterized protein DDB_G0272718-like | **6.77** |
| XM_001344665 | si:dkey-204a24.10 | si:dkey-204a24.10 | **6.64** |
| NM_213639 | cfl1l | cofilin 1 (non-muscle), like | **6.60** |
| XM_021481194 | si:dkeyp-11e3.1 | si:dkeyp-11e3.1 | **6.56** |
| NM_001005603,XM_005162569 | gcm2 | glial cells missing homolog 2 (Drosophila) | **6.53** |
| NM_001305565 | styk1 | serine/threonine/tyrosine kinase 1 | **6.51** |
| XM_001922682 | abcb5 | ATP-binding cassette, sub-family B (MDR/TAP), member 5 | **6.48** |
| XM_017351113 | si:zfos-411a11.2 | si:zfos-411a11.2 | **6.27** |
| NM_001159512 | tmprss13a | transmembrane protease, serine 13a | **6.22** |
| XM_009306880,XM_017358564,XM_017358565,XM_017358566 | si:ch211-39f2.3 | si:ch211-39f2.3, transcript variant X1 | **6.16** |
| NM_001003753 | gna14 | guanine nucleotide binding protein (G protein), alpha 14 | **6.13** |
| XM_701335 | lye | lymphocyte antigen-6, epidermis | **6.10** |
| XM_021478122 | LOC108190590 | cell wall protein DAN4-like | **6.06** |
| XM_017351848 | si:ch211-202f3.3 | si:ch211-202f3.3 | **5.98** |
| XM_690939 | si:ch211-241e1.3 | si:ch211-241e1.3 | **5.87** |
| XM_001337411 | si:ch211-95j8.2 | si:ch211-95j8.2 | **5.85** |
| NM_001177333 | ugt2a4 | UDP glucuronosyltransferase 2 family, polypeptide A4 | **5.62** |
| XM_005166636 | LOC110437729 | uncharacterized LOC110437729 | **5.53** |
| NM_200512,XM_005163733 | cyp2k18 | cytochrome P450, family 2, subfamily K, polypeptide 18 | **5.48** |
| XM_017355793 | LOC101886478 | uncharacterized LOC101886478 | **5.47** |
| NM_152884 | caspb | caspase b | **5.38** |
| XM_003200112 | LOC100332535 | uncharacterized LOC100332535 | **5.38** |
| XR_001796808,XR_002456261,XR_002456262,XR_002456264,XR_002456265,XR_659263 | LOC103909381 | uncharacterized LOC103909381, transcript variant X5 | **5.25** |

| | | | |
|---|---|---|---|
| XM_017354704 | LOC100334219 | GTPase IMAP family member 8 | **5.25** |
| XM_005174119,XR_002456555 | si:ch211-200e2.1 | si:ch211-200e2.1, transcript variant X2 | **5.23** |
| NM_194418 | sepw2b | selenoprotein W, 2b | **5.23** |
| XM_002666100 | elf3 | E74-like factor 3 (ets domain transcription factor, epithelial-specific) | **5.13** |
| NM_001123324,XM_005167145,XM_021478158 | zgc:194839 | zgc:194839 | **5.12** |
| XM_021479509 | hephl1b | hephaestin-like 1b, transcript variant X1 | **5.09** |
| XM_682484 | dok1a | docking protein 1a | **5.08** |
| NM_001080700,XM_005157590,XM_005157591 | aifm4 | apoptosis-inducing factor, mitochondrion-associated, 4 | **5.06** |
| XM_021467427,XM_021467428,XM_021467429 | grhl3 | grainyhead-like transcription factor 3, transcript variant X1 | **5.05** |
| XR_002459322 | LOC110439977 | uncharacterized LOC110439977 | **5.05** |
| XR_002457354,XR_002457355,XR_002457356 | si:ch73-264i18.3 | si:ch73-264i18.3, transcript variant X1 | **5.04** |
| XM_021468516 | LOC101886665 | growth/differentiation factor 15, transcript variant X1 | **5.04** |
| XM_692747 | tnfaip2b | tumor necrosis factor, alpha-induced protein 2b | **5.03** |
| NR_029993,rna-mir1-1 | mir1-1 | microRNA 1-1 | **-13.23** |
| XM_001331878 | tgm1l3 | transglutaminase 1 like 3 | **-12.38** |
| NR_134975 | si:ch211-255g12.6 | si:ch211-255g12.6 | **-12.21** |
| NM_001130626,XM_005163024 | kcna6a | potassium voltage-gated channel, shaker-related, subfamily, member 6 a | **-12.05** |
| NM_152950 | cryaa | crystallin, alpha A | **-11.12** |
| NM_001033722 | crybgx | crystallin beta gamma X | **-11.07** |
| XM_021474277,XM_021474281 | LOC100007086 | prostatic spermine-binding protein-like, transcript variant X2 | **-10.93** |
| XM_001345063,XM_009303924 | gls2a | glutaminase 2a (liver, mitochondrial), transcript variant X2 | **-10.83** |
| XM_685653,XR_002456229 | slc10a1 | solute carrier family 10 (sodium/bile acid cotransporter), member 1, transcript variant X2 | **-10.61** |
| NM_001102388 | ankrd1b | ankyrin repeat domain 1b (cardiac muscle) | **-9.71** |
| XM_001921697,XM_021477806 | LOC100147904 | zonadhesin, transcript variant X1 | **-9.70** |
| XM_009294870 | si:dkey-1j5.4 | si:dkey-1j5.4 | **-9.41** |
| NM_001007058 | crygm5 | crystallin, gamma M5 | **-9.18** |
| rna-trnS2 | trnS2 | tRNA-Ser | **-8.99** |
| XM_686705 | klhl38b | kelch-like family member 38b | **-8.94** |
| XM_009295578,XM_021469189,XM_021469190,XM_021469191,XR_002456370 | phka1b | phosphorylase kinase, alpha 1b (muscle), transcript variant X1 | **-8.79** |
| NM_001123311 | si:dkey-57a22.15 | si:dkey-57a22.15 | **-8.41** |
| NM_001145593 | si:ch211-89o9.4 | si:ch211-89o9.4 | **-8.37** |
| NR_030507,rna-mir726 | mir726 | microRNA 726 | **-8.13** |
| NM_001002408 | crygm2d13 | crystallin, gamma M2d13 | **-7.95** |

| | | | |
|---|---|---|---|
| NM_001002582 | crygm2d15 | crystallin, gamma M2d15 | **-7.67** |
| NM_001099452 | hspb3 | heat shock protein, alpha-crystallin-related, b3 | **-7.64** |
| NM_001099243 | crygm2d17 | crystallin, gamma M2d17 | **-7.42** |
| NM_001044863 | crygm2d5 | crystallin, gamma M2d5 | **-7.30** |
| NM_001083825,XM_005173960,XR_002456414 | gls2b | glutaminase 2b (liver, mitochondrial) | **-7.25** |
| NM_001123318 | crygm2d9 | crystallin, gamma M2d9 | **-7.20** |
| NM_001109860 | crygm2d10 | crystallin, gamma M2d10 | **-7.15** |
| NM_001123293 | crygm2d21 | crystallin, gamma M2d21 | **-7.12** |
| XM_001341714 | si:dkey-8e10.3 | si:dkey-8e10.3 | **-6.95** |
| XM_001337585 | si:ch1073-70f20.1 | si:ch1073-70f20.1 | **-6.93** |
| XM_021478899 | crygm2d19 | crystallin, gamma M2d19 | **-6.92** |
| XM_001920198,XM_021477931 | LOC560010 | uncharacterized LOC560010, transcript variant X1 | **-6.92** |
| NM_001109858 | crygm2d11 | crystallin, gamma M2d11 | **-6.87** |
| NM_001002142 | crygm2d18 | crystallin, gamma M2d18 | **-6.85** |
| NM_001327912,XM_005169486,XM_021480169 | si:ch73-111m19.2 | si:ch73-111m19.2 | **-6.78** |
| NM_001082939 | crygm2d20 | crystallin, gamma M2d20 | **-6.77** |
| NM_001045107 | crygm2d7 | crystallin, gamma M2d7 | **-6.70** |
| NM_001002581 | crygm2d8 | crystallin, gamma M2d8 | **-6.64** |
| NM_001080061 | crygm2d3 | crystallin, gamma M2d3 | **-6.43** |
| NM_001089431 | crygm2d4 | crystallin, gamma M2d4 | **-6.42** |
| NM_200614 | hsc70 | heat shock cognate 70 | **-6.36** |
| NM_001002584,XM_005167477 | cryba2b | crystallin, beta A2b | **-6.33** |
| XM_021481258 | map1lc3cl | microtubule-associated protein 1 light chain 3 gamma, like | **-6.30** |
| NM_001103134 | crygm2d16 | crystallin, gamma M2d16 | **-6.25** |
| NM_001039825,XM_017358479 | scn4aa | sodium channel, voltage-gated, type IV, alpha, a | **-6.22** |
| rna-trnH | trnH | tRNA-His | **-6.15** |
| NM_001204332 | gngt2b | guanine nucleotide binding protein (G protein), gamma transducing activity polypeptide 2b | **-6.14** |
| NM_001326385 | cd59 | CD59 molecule (CD59 blood group) | **-6.13** |
| NM_001013262 | crygmx | crystallin, gamma MX | **-6.11** |
| NM_001044866 | crygm2d2 | crystallin, gamma M2d2 | **-6.07** |
| XM_001340280,XM_002665557,XM_002665561,XM_002665562,XM_005170079,XM_005170080,XM_005170081,XM_005170082,XM_021468312 | mbpa | myelin basic protein a, transcript variant X1 | **-6.07** |
| XR_002459186 | LOC108190625 | uncharacterized LOC108190625 | **-5.99** |
| NM_182891 | opn1mw2 | opsin 1 (cone pigments), medium-wave-sensitive, 2 | **-5.96** |
| NR_030060,rna-mir124-2,rna-mir124-2-2 | mir124-2 | microRNA 124-2 | **-5.96** |

| | | | |
|---|---|---|---|
| NM_001045282 | zgc:136461 | zgc:136461 | **-5.86** |
| XM_005170278 | tusc5b | tumor suppressor candidate 5b | **-5.85** |
| NM_001102394 | crygm2d14 | crystallin, gamma M2d14 | **-5.84** |
| NM_001020681 | crygm2d1 | crystallin, gamma M2d1 | **-5.70** |
| XM_689410 | cthrc1b | collagen triple helix repeat containing 1b | **-5.67** |
| NM_001199870,XM_005173089 | loxl3a | lysyl oxidase-like 3a | **-5.65** |
| NM_001017738 | cryba1l1 | crystallin, beta A1, like 1 | **-5.65** |
| NM_199967 | gngt1 | guanine nucleotide binding protein (G protein), gamma transducing activity polypeptide 1 | **-5.53** |
| NM_001003428 | crygn2 | crystallin, gamma N2 | **-5.51** |
| NR_030079,rna-mir130c-2,rna-mir130c-2-2 | mir130c-2 | microRNA 130c-2 | **-5.51** |
| NM_001024411 | pbld | phenazine biosynthesis like protein domain containing | **-5.46** |
| NM_001114848 | si:dkey-245p14.4 | si:dkey-245p14.4 | **-5.39** |
| NM_001024427 | crygmxl2 | crystallin, gamma MX, like 2 | **-5.34** |
| XM_001921984 | soul5 | heme-binding protein soul5 | **-5.33** |
| NM_205700 | cfl2 | cofilin 2 (muscle) | **-5.31** |
| XR_002459141 | LOC101884156 | uncharacterized LOC101884156 | **-5.29** |
| XM_005163153 | prr33 | proline rich 33 | **-5.29** |
| NM_131319 | opn1sw1 | opsin 1 (cone pigments), short-wave-sensitive 1 | **-5.28** |
| NM_001045007,XM_005169953 | si:ch211-219a15.3 | si:ch211-219a15.3 | **-5.25** |
| NM_001020668 | zgc:112242 | zgc:112242 | **-5.23** |
| NM_001013297 | prnpb | prion protein b | **-5.23** |
| XR_002456662 | LOC110438368 | uncharacterized LOC110438368 | **-5.17** |
| NM_214702 | slc25a4 | solute carrier family 25 (mitochondrial carrier; adenine nucleotide translocator), member 4 | **-5.13** |
| NM_001002586 | cryba1b | crystallin, beta A1b | **-5.13** |
| XM_021469185 | prob1 | proline-rich basic protein 1 | **-5.12** |
| XM_002662316 | si:ch73-23l24.1 | si:ch73-23l24.1 | **-5.11** |
| XM_009296766 | asb14b | ankyrin repeat and SOCS box containing 14b | **-5.09** |
| NR_045230,rna-mir143-2 | mir143-2 | microRNA 143-2 | **-5.08** |
| NM_001002049 | cryba2a | crystallin, beta A2a | **-5.08** |
| NM_001020674 | gda | guanine deaminase | **-5.05** |
| NM_131192 | opn1sw2 | opsin 1 (cone pigments), short-wave-sensitive 2 | **-5.05** |
| XM_021466343 | LOC110437898 | uncharacterized LOC110437898 | **-5.01** |
