# Supplementary figures and images for: Transcriptome-Based Identification of Genes Responding to the Organophosphate Pesticide Phosmet in Danio rerio
Source: Genes (Basel). 2021 Oct 29;12(11):1738. doi: 10.3390/genes12111738 (PMC8624534; doi:10.3390/genes12111738)

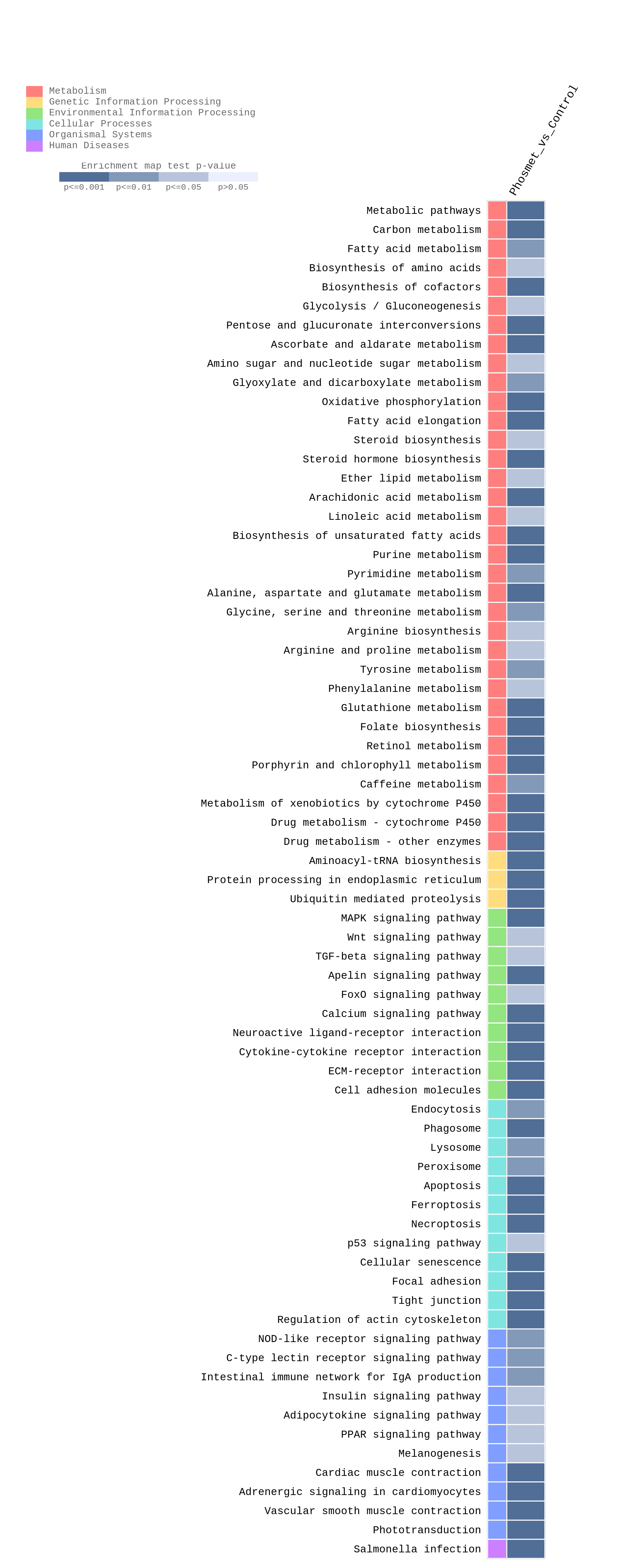

Supplement: Supplementary file 1 [file genes-12-01738-s001.zip › genes-1403670-supplementary/Supplementary material/Figure S1.png]
